# SET domain containing protein 5 (SETD5) enhances tumor cell invasion and is associated with a poor prognosis in non-small cell lung cancer patients

**DOI:** 10.1186/s12885-019-5944-2

**Published:** 2019-07-25

**Authors:** Hairu Yu, Jiayi Sun, Congxuan Zhao, Haotian Wang, Yeqiu Liu, Jiajia Xiong, Jing Chang, Mixue Wang, Wenhui Wang, Dongman Ye, Hongyan Zhou, Tao Yu

**Affiliations:** 1 Department of Medical Imaging, Cancer Hospital of China Medical University, No. 44 Xiaoheyan Road, Dadong District, Shenyang, 110042 Liaoning Province China; 2 Department of Medical Imaging, Liaoning Cancer Hospital and Institute, No. 44 Xiaoheyan Road, Dadong District, Shenyang, 110042 Liaoning Province China; 3 The First Clinical College, Dalian Medical University, No. 9 West Section of Lushun South Road, Dalian City, Liaoning Province China

**Keywords:** SET domain containing 5 (SETD5), Non-small cell lung cancer, Invasion, ERK signaling, Prognosis

## Abstract

**Background:**

SET domain containing 5 (SETD5) is related to the aggressiveness of prostate and mammary cancers, but its association with non-small cell lung cancer (NSCLC) is unknown. Therefore, the purpose of this research was to determine the expression pattern and function of SETD5 in NSCLC.

**Methods:**

SETD5 was detected by immunohistochemical analysis in 147 patients with non-small cell lung cancer. SETD5 was overexpressed in A549 cells or suppressed with siRNA in H1299 cells. Wound healing and transwell assays were performed. The expression levels of SETD5, p-AKT/AKT, Snail, p-JNK/JNK, Slug, E-cadherin, Zo-1, p-P38/P38, occludin, α-catenin, p-ERK/ERK, and p-P90RSK/ P90RSK were assessed by western blot.

**Results:**

Online analysis of overall survival in 1928 patients with NSCLC showed that the SETD5 gene was related to worse overall survival (OS)(*P* < 0.001). The positive expression rate of SETD5 in noncancerous tissues was lower than that in cancerous tissues (16.7% vs. 44.2%, *P* < 0.001). SETD5 was significantly correlated with advanced TNM stage (*P* < 0.001), lymph node metastasis (*P* < 0.001) and overall survival rate (*P* < 0.001). Overexpression of SETD5 in A549 cells increased migration and invasion, while deletion of SETD5 in H1299 cells decreased migration and invasion. After overexpression of SETD5, the expression of ZO-1 was downregulated, and that of Snail was upregulated. After overexpression of SETD5, the levels of p-ERK and its downstream factor p-p90rsk increased.

**Conclusion:**

These results suggest that SETD5 could regulate p-P90RSK and facilitate the migration and invasion of NSCLC and may be related to the poor prognosis of patients with NSCLC.

## Background

Non-small cell lung cancer (NSCLC) is a malignant tumor of the lung accounting for 85–90% of all lung cancers [1]. It affects mainly adults > 65 years of age, men, and tobacco smokers [1, 2]. In the USA, the incidence of NSCLC is 75 per 100,000 men and 53.5 per 100,000 women [3]. Mortality is high, with 55.9 per 100,000 men and 36.3 per 100,000 women [3]. The treatment for NSCLC is multidisciplinary and includes surgery, chemotherapy, and radiation therapy [2]. Despite great advances in techniques, regimens, and targeted therapies, the 5-year survival for patients with NSCLC (all stages together) is only 18% [4], highlighting the need to better understand the disease to further improve the treatment strategies.

SETD5 (SET domain containing 5), localized on chromosome 3p25.3, is a member of the SET domain protein family. These proteins play pivotal roles in histone lysine methylation, thus inducing numerous cellular processes, including heterochromatin formation, X-chromosome inactivation, and transcription regulation [5, 6]. Osipovich et al. [7] also found that SETD5 plays an important role in the co-transcriptional regulation of mammalian development and histone acetylation. Previous studies demonstrated that SET domain family proteins exhibited diverse biological roles in cancer progression [8–17]. Nevertheless, the expression pattern and biological roles of SETD5 in human malignant cancers remain unclear. Kuechler et al. [18] confirmed that loss of function of SETD5 was associated with intellectual disability and was the critical driver of the phenotype of 3p25.3 microdeletion syndrome [18–20]. Poissonnier et al. [21] showed that miR126-5p abolished leukocyte transendothelial migration by suppressing SETD5, indicating that SETD5 may participate in the process of migration and invasion. A microarray analysis suggested that the SETD5 locus was associated with prostate cancer aggressiveness [22]. A transcriptomics study also showed that SETD5 was associated with the treatment reaction in metastatic prostate tumors [23]. High mRNA levels of SETD5 were related to poor prognosis in patients with breast tumors [24]. Nevertheless, studies directly assessing the mechanistic role of SETD5 in tumors are lacking.

Therefore, the objective of this research was to determine the expression pattern and function of SETD5 in NSCLC. The results showed that SETD5 enhanced the invasion of NSCLC cells by activating the ERK signaling pathway, suggesting that SETD5 may be a therapeutic target for NSCLC patients.

## Methods

### Online analysis of the total survival rate in patients with NSCLC

To assess the relationship between the expression of SETD5 and patient clinical results, we used the KM Plotter Online Tool for NSCLC patients (http://www.kmplot.com). This is a public database with information about 1928 patients that allows us to examine the relevance of genes with overall survival (OS). The clinical features of all specimens have been described [25].

### Patients and clinical specimens

Tissue samples were obtained from 147 patients who underwent complete surgical excision at the Cancer Hospital of China Medical University from 2009 to 2011. All specimens were diagnosed as lung squamous cell carcinoma or lung adenocarcinoma. No patients had received chemotherapy or neoadjuvant radiotherapy, and all patients received chemotherapy after surgery. Adjuvant chemotherapy was started from 3 to 4 weeks after the operation. The chemotherapy regimen was as follows: NP, GP regimen or according to drug sensitive gene test results. In principle, a platinum-containing two-drug regimen should be applied. The chemotherapy cycle was generally 4–6 cycles. Of the 147 patients, 48 had corresponding non-cancerous tissues available. All patients were followed up. NSCLC-specific survival was defined as the time from surgery to the end of follow-up or death due to relapse or transfer [19]. Histological diagnosis and grading were assessed according to the World Health Organization (WHO) classification of lung tumors from 2015 [26]. Tumor staging was based on the seventh edition of the International Union against Cancer (UICC) TNM Staging System for Lung Cancer [27]. The characteristics of the cases and cancers are presented in Table 1. The research was approved by the Institutional Review Committee of China Medical University. Informed consent was obtained from each patient to use their specimens for research purposes. Written consent was provided in the ethics approval and consent to participate section.

**Table 1:** Correlations between SETD5 expression and clinicopathological features in non-small cell lung cancer (NSCLC)

Clinical parameters	Number (*N* = 147)	SETD5 expression		χ2	*P*
		Positive	Negative		
Age (years)				0.573	0.449
< 59	65	35	30		
≥ 59	82	39	43		
Gender				1.551	0.213
Male	88	48	40		
Female	59	26	33		
Histological type				0.993	0.609
Squamous cell carcinoma	54	27	27		
Adenocarcinoma	92	46	46		
Large cell carcinoma	1	1	0		
Differentiation				0.329	0.566
Well	57	27	30		
Moderate + Poor	90	47	43		
TNM stages				12.590	< 0.001
I + II	103	42	61		
III	44	32	12		
Lymph node metastasis				15.252	< 0.001
Positive	66	45	21		
Negative	81	29	52		

*TNM* tumor node metastasis

### Immunohistochemistry (IHC)

Samples were fixed in 10% neutral formalin, embedded in paraffin (Shanghai Shenggong Biological Engineering Co., Ltd., Shanghai, China), and sectioned at 4 μm. IHC was performed using the streptavidin-peroxidase method. Tissue slices were incubated with a polyclonal rabbit anti-SETD5 antibody (1,100, ab139987; Abcam, Cambridge, UK) at 4 °C overnight; then, we used a biotin goat anti-mouse IgG secondary antibody (Ultrasensitive; MaiXin, Fuzhou, China). After washing, the tissue slices were incubated with horseradish peroxidase binding streptomycin biotin (Ultrasensitive; MaiXin, Fuzhou, China), and 3,3-diaminobenzidine tetrachloride (MaiXin, Fuzhou, China) was used for development. Finally, the samples were lightly re-dyed with hematoxylin (Shanghai Shenggong Biological Engineering Co., Ltd., Shanghai, China), dehydrated and fixed in alcohol. Without considering the clinical data, the two researchers semi-quantitatively scored the slides by assessing the staining intensity and percentage of stained cells in representative areas. The staining intensity was scored as 0 (not stained), 1 (weak), 2 (moderate), or 3 (strong). The percentage of stained cells was scored as 1 (1–25%), 2 (26–50%), 3 (51–75%), or 4 (76–100%). Finally, the intensity and percentage scores were multiplied to obtain 0–12 points. A score ≥ 4 proved that the tumors were positive for SETD5 expression. Tumor specimens scoring between 1 and 3 were classified as having weak expression, while those scoring 0 were considered to have no expression; both weak expression and no expression were defined as negative SETD5 expression.

### Cell culture

The HBE cell line was obtained from the American Type Culture Collection (ATCC; Manassas, VA, USA). The H1299, H460, A549, H292, and SK-MES-1 cell lines were purchased from the Shanghai Cell Bank (Shanghai, China). All of these cells were cultured in RPMI 1640 (Invitrogen, Carlsbad, CA, USA) containing 10% fetal bovine serum (Invitrogen, Carlsbad, CA, USA), 100 μg/ml streptomycin (Sigma, St Louis, MO, USA), and 100 IU/ml penicillin (Sigma, St Louis, MO, USA). Cells were passaged every other day using 0.25% trypsin (Invitrogen, Carlsbad, CA, USA).

### Plasmid transfection and small interfering RNA treatment

We bought the pCMV6-ddk-myc-SETD5 and pCMV6-ddk-myc plasmids from Origene (RC240118, Rockville, MD, USA). SETD5-siRNA (sc-78478) and NC-siRNA (sc-37007) were obtained from Santa Cruz Biotechnology (Santa Cruz, CA, USA). Transfection was carried out using the Lipofectamine 3000 reagent (Invitrogen, Carlsbad, CA, USA) according to the manufacturer’s instructions.

### Wound healing assay

Wounds were created in confluent areas of cell monolayers with < 90% confluence 48 h after transfection using a 200-μl pipette tip. Cell migration into the wound areas at different time points was observed. ImageJ software (National Institutes of Health, Bethesda, MD, USA) was used to measure the distance the cells traveled into the wound areas. Representative images were captured. Each specimen was analyzed twice, and three independent experiments were carried out.

### Matrigel invasion assay

Cell invasion assays were carried out in 24-well Transwell chambers with 8-μm pores (Costar, Cambridge, MA, USA). The inserts were coated with 20 μl of Matrigel in RPMI 1640 medium (1:3; BD Bioscience, San Jose, CA, USA). Cells were trypsinized 48 h after transfection, resuspended at 3 × 10^5^ cells in 100 μl of serum-free medium, and transferred to the upper transwell chamber; 10% FBS was added to the lower chamber as a chemoattractant. After incubation for 18 h, cells that passed through the filter were fixed with 4% paraformaldehyde and stained with hematoxylin (Zhongshan Jinqiao Biotechnology Co., Ltd., Beijing, China). Next, we randomly selected 10 visual fields at 40× magnification under a microscope (Leica Microsystems, Wetzlar, Germany) and counted the number of cells that invaded the subventricular space.

### Western blotting

Protein was extracted with a lysis buffer (Pierce, Rockford, IL, USA) and quantified with the Bradford method [28]. We used 10% sodium dodecyl sulfate-polyacrylamide gel electrophoresis to isolate the proteins (50 μg) and transferred them to polyvinylidene fluoride (PVDF; Millipore, Billerica, MA, USA) membranes. We incubated the membranes overnight at 4 °C with the following primary antibodies: SETD5 (1:100, ab139987; Abcam, Cambridge, UK); GAPDH (1:5000, Sigma, St Louis, MO, USA); Myc-tag, Snail, Slug, p-P38, P38, p-ERK, ERK, p-AKT, AKT, p-JNK, JNK, p-P90RSK, P90RSK (1:1000; Cell Signaling Technology, Danvers, MA, USA); α-catenin (1:500; BD Transduction Laboratories, Lexington, KY, USA); Zo-1, E-cadherin (1:1000; BD Transduction Laboratories, Lexington, KY, USA); and occludin (1:500; Proteintech, Chicago, IL, USA). Next, we washed the membranes and incubated them with peroxidase-bound anti-rat or anti-rabbit IgG (Santa Cruz Biotechnology, Santa Cruz, CA, USA) at 37 °C for 2 h. We visualized the proteins by electrochemiluminescence (Pierce, Rockford, IL, USA) and detected them with a bio-imaging system (DNR Bio-Imaging Systems, Jerusalem, Israel).

### Statistical analysis

All our data analyses were performed using SPSS22.0 for Windows (IBM, Armonk, NY, USA). To evaluate the correlations between SETD5 and clinicopathological factors, the Pearson Chi-square test was used. Kaplan-Meier survival analyses were performed, and curves were compared using the log-rank test. To estimate prognostic factors, we used the Cox regression model for univariate and multivariate analysis. We used the Mann-Whitney U test to analyze the results of the invasion assay. *P* < 0.05 was considered to have statistical significance.

## Results

### SETD5 is related to worse overall survival in 1928 NSCLC patients from a public database

**Fig. 1:**
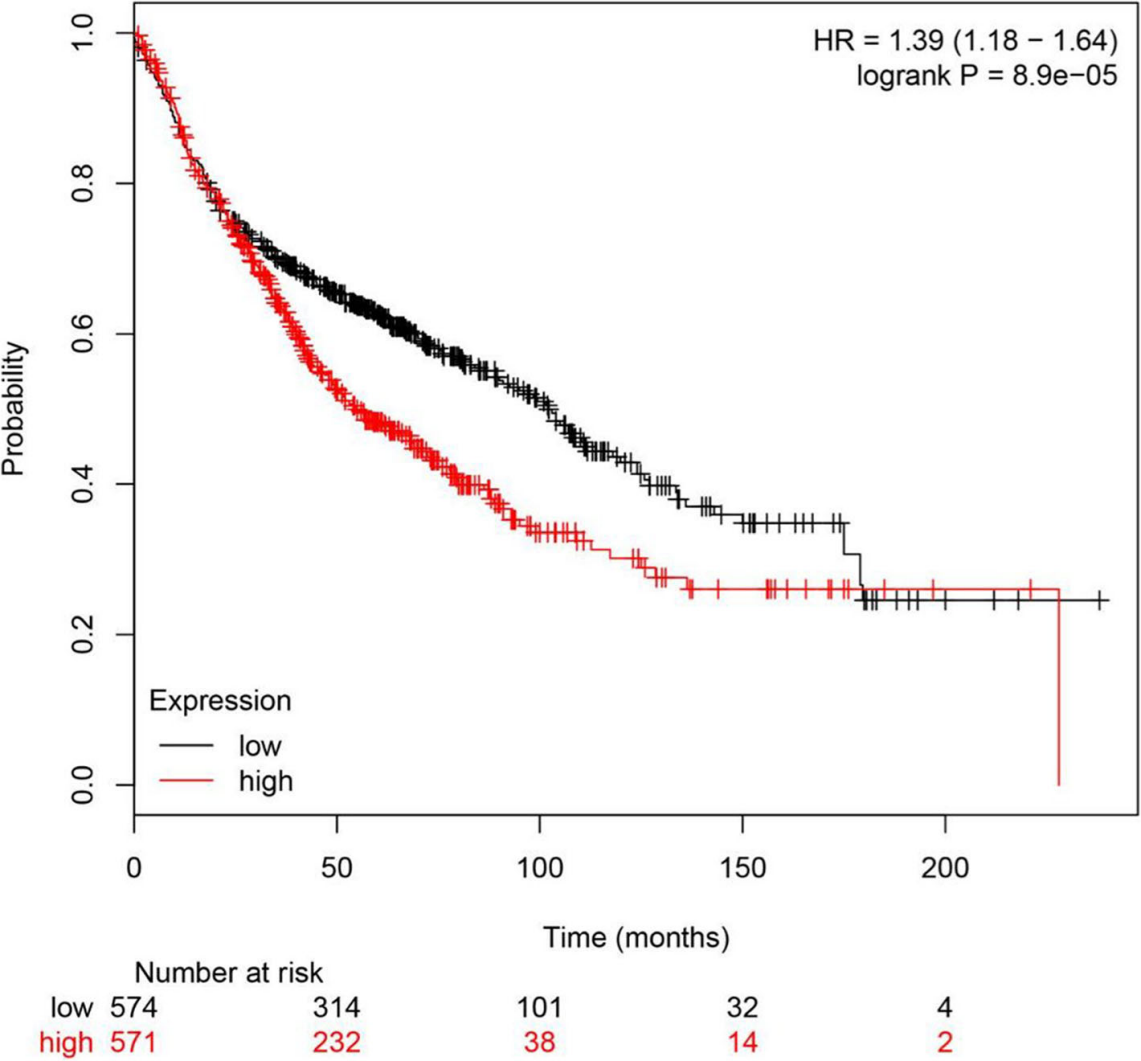
Online analysis of the overall survival of 1928 patients with NSCLC. The relationship between SETD5 expression and overall survival was evaluated using the KM Plotter Online Tool in 1928 patients with NSCLC. NSCLC, non-small cell lung cancer; HR, hazard ratio

To preliminarily examine the potential role of SETD5 in NSCLC, the online tool KM plotter was used to predict the effect of SETD5 gene expression on OS in 1928 patients with NSCLC. As shown in Fig. 1, the SETD5 gene was related to worse OS in patients with NSCLC (*p* < 0.001).

### SETD5 was upregulated in NSCLC and is related to poor prognosis in NSCLC patients

**Fig. 2:**
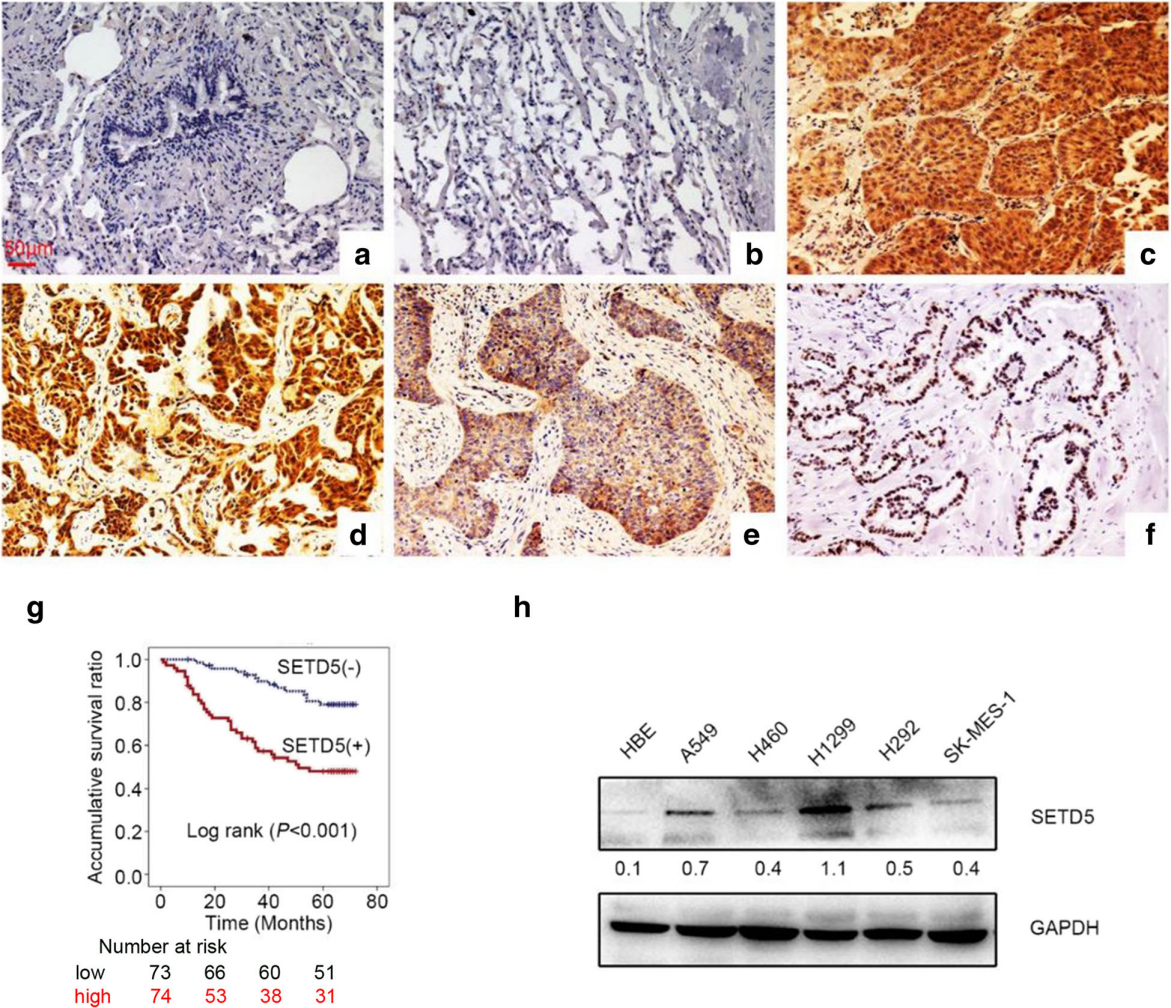
SETD5 expression in NSCLC specimens and cell lines. **a**-**f** Representative SETD5 expression in adjacent normal tissues, squamous cell carcinoma tissues, and adenocarcinoma tissues detected by immunohistochemistry. **a** Normal bronchial tissue, **b** alveolar epithelial tissue, **c** squamous cell carcinoma, and **d** adenocarcinoma, only localized in the cytoplasm (**e**) or the nuclei (**f**) in some cases. Scale bar = 50 μm. **g** Kaplan-Meier analysis of the association between SETD5 expression and overall survival in patients with NSCLC. **h** SETD5 expression in different NSCLC cell lines detected by western blot. GAPDH was used as an internal control

Next, to prove the results from the KM plotter tool, we performed IHC on 147 specimens of NSCLC and 48 specimens of corresponding normal lung tissues to detect the expression and subcellular localization of SETD5. The expression of SETD5 was low in peritumoral lung tissues (Fig. 2a-b) but high in the cytoplasm and nuclei of NSCLC specimens (Fig. 2c-d). The positive expression rate of SETD5 in peritumoral normal tissues (8/48) was lower than that in cancerous tissues (65/147) (16.7% vs. 44.2%, *P* < 0.001). In a few cases, we found that SETD5 was localized only in the cytoplasm (5.4%, 8/147, Fig. 2e) or the nuclei (3.4%, 5/147, Fig. 2f).

Positive expression of SETD5 was significantly associated with advanced TNM stage (*P* < 0.001) and lymph node metastasis (*P* < 0.001) but not with age, sex, histological type, or differentiation (all *P* > 0.05, Table 1). A Kaplan-Meier analysis showed that the OS was shorter in patients with positive SETD5 expression than in those with negative SETD5 expression (46.8 ± 3.1 vs. 64.9 ± 1.8 months, *P* < 0.001, Fig. 2g). Through univariate analysis (UA) and multivariate analysis (MA), we concluded that along with positive lymph node metastasis (*P* < 0.001 for UA and *P* = 0.012 for MA), the independent prognostic factors of OS in NSCLC patients may be related to the SETD5 overexpression (*P* < 0.001 for UA and *P* = 0.013 for MA, Table 2). Then, we assessed the SETD5 protein levels in various NSCLC cell lines and the human bronchial epithelial cell line HBE by western blot. The results showed that the expression of SETD5 in HBE cells was lower than that in NSCLC cell lines (Fig. 2h). Therefore, we can conclude that SETD5 is likely to play an important role in NSCLC.

**Table 2:** Univariate and multivariate analyses of the associations between clinicopathological features and overall survival in NSCLC patients

Variables	Hazard ratio	*P*
	(95% CI)	
Univariate analysis		
Age	0.795 (0.458–1.378)	0.413
Gender	0.997 (0.571–1.742)	0.992
Histological type	1.539 (0.852–2.778)	0.153
Differentiation	1.989 (1.075–3.682)	0.029
TNM stages	5.274 (2.983–9.324)	< 0.001
Lymph node metastasis	6.415 (3.338–12.326)	< 0.001
SETD5 expression	3.493 (1.886–6.473)	< 0.001
Multivariate analysis		
Differentiation	1.425 (0.757–2.683)	0.273
TNM stages	1.981 (0.953–4.116)	0.067
Lymph node metastasis	3.034 (1.272–7.233)	0.012
SETD5 expression	2.267 (1.192–4.311)	0.013

### SETD5 enhanced NSCLC cell migration and invasion

**Fig. 3:**
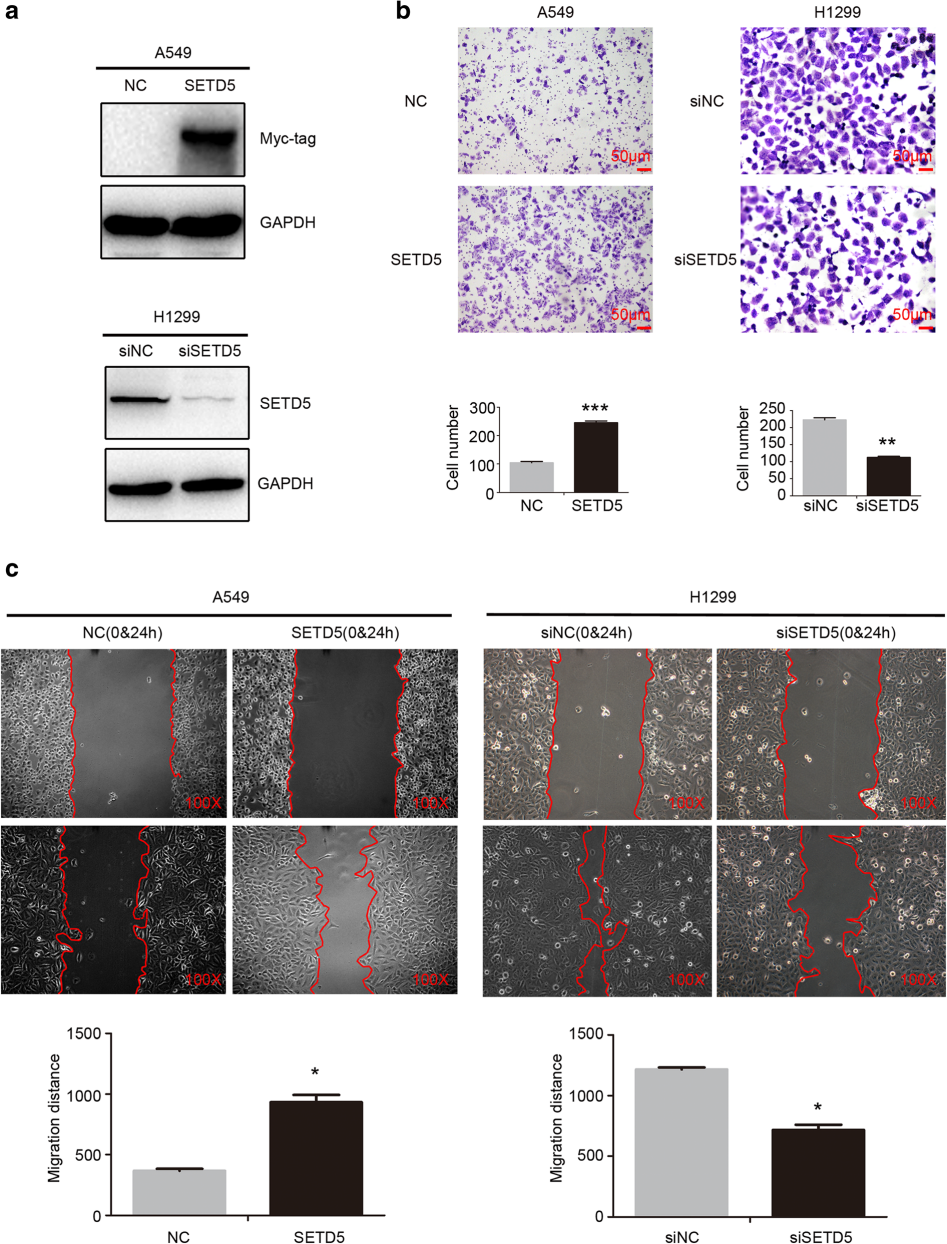
SETD5 promoted the migration and invasion of NSCLC cells. **a** Western blot analysis of SETD5 protein levels after SETD5 overexpression in A549 cells or SETD5 silencing in H1299 cells. **b** Cell migration was assessed by wound healing assay after SETD5 overexpression in A549 cells or SETD5 knockdown in H1299 cells. **c** Invasion was detected using transwell assays after SETD5 overexpression in A549 cells or SETD5 knockdown in H1299 cells. Scale bar = 50 μm. The data are shown as the mean ± standard deviation (SD) from three independent experiments. *P < 0.05; **P < 0.01; ***P < 0.001

To better understand the role of SETD5 in NSCLC aggressiveness, we overexpressed or suppressed SETD5 in A549 or H1299 cells, respectively (Fig. 3a). Through wound healing and transwell assays, we revealed that migration (Fig. 3b) and invasion (Fig. 3c) increased after overexpressing SETD5 in A549 cells. Migration (Fig. 3b) and invasion (Fig. 3c) were decreased after depleting SETD5 in H1299 cells. Hence, these results suggest that SETD5 expression plays a role in the aggressiveness of NSCLC.

### SETD5 promoted ERK and P90RSK phosphorylation, upregulated snail and downregulated zo-1

**Fig. 4:**
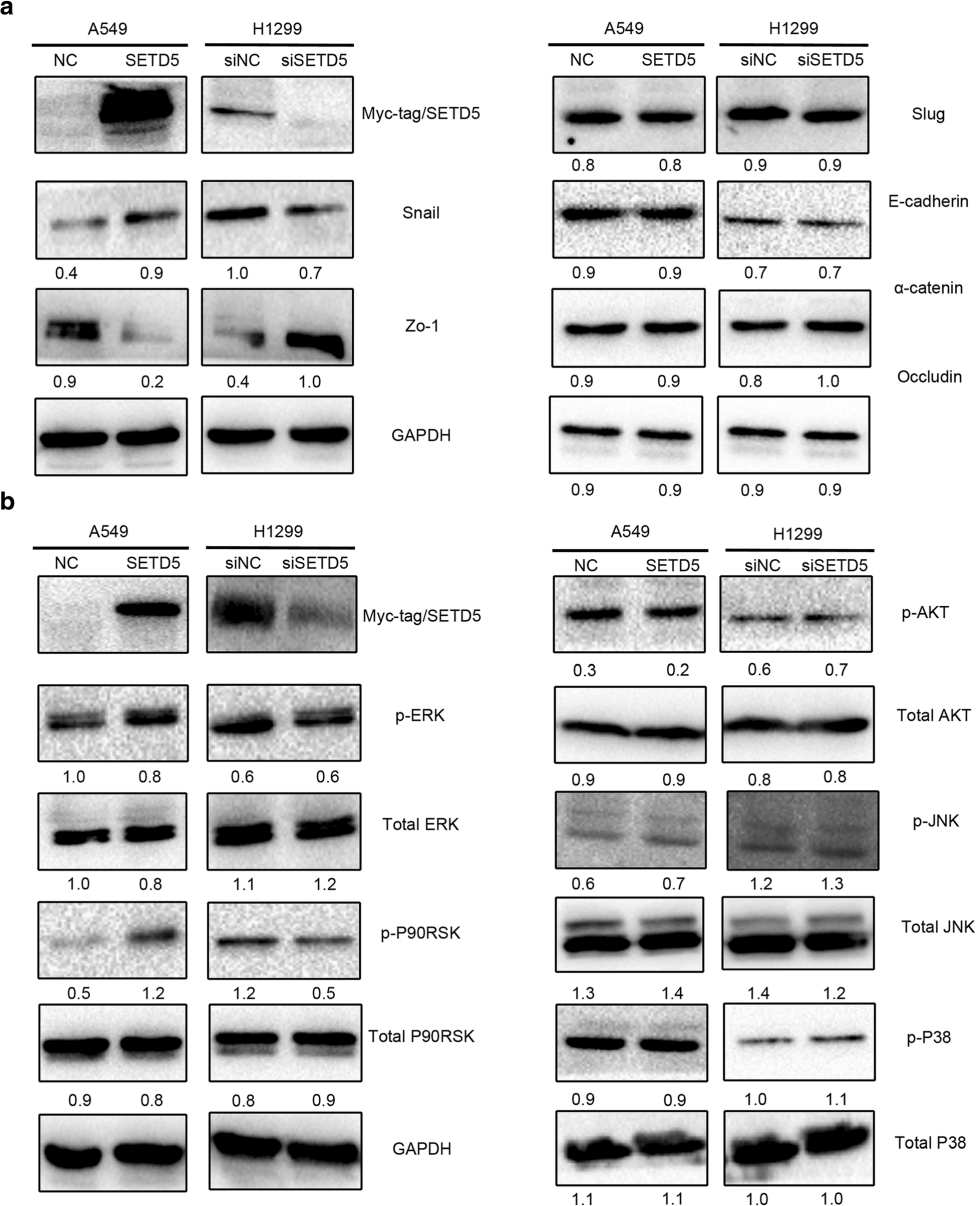
Overexpression of SETD5 upregulated p-ERK, p-P90RSK, and Snail and downregulated Zo-1 in NSCLC cells. SETD5 was overexpressed in A549 cells or suppressed with siRNA in H1299 cells. **a** EMT-related proteins were measured by western blot. **b** MAPK-related proteins were measured by western blot. GAPDH was used as an internal control. EMT, epithelial-mesenchymal transition; MAPKs, mitogen-activated protein kinases

Finally, to explore the possible mechanisms involved in the regulation of NSCLC aggressiveness by SETD5, we screened epithelial-mesenchymal transition (EMT)-related proteins and key signaling pathway proteins. Regarding EMT-related proteins, western blot results suggested that Snail was upregulated and that Zo-1 was downregulated when SETD5 was overexpressed in A549 cells. Snail and Zo-1 were downregulated after silencing SETD5 with siRNA (Fig. 4a). Slug, E-cadherin, α-catenin, and occludin were unchanged (Fig. 4a).

Regarding key cell proliferation pathways, western blot results indicated that p-ERK and its downstream factor p-P90RSK were enhanced after overexpressing SETD5 in A549 cells, while p-ERK and P90RSK were decreased after SETD5 inhibition via siRNA in H1299 cells (Fig. 4b). The levels of p-P38, P38, p-AKT, AKT, p-JNK, and JNK showed no obvious alterations (Fig. 4b). These results suggest that SETD5 may facilitate NSCLC cell invasion by promoting the phosphorylation of ERK and P90RSK and then upregulating Snail and downregulating Zo-1.

## Discussion

SETD5 plays a key role in mammalian development and histone acetylation co-transcription. SETD5 is a member of the SET domain protein family [5–7]. SETD5 is related to the aggressiveness of prostate and mammary cancers [22–24], but the mechanism of its role in non-small cell lung cancer remains unclear. This study showed that SETD5 was significantly correlated with lymph node metastasis, advanced TNM stage and OS in NSCLC patients. SETD5 may promote the migration and invasion of NSCLC. SETD5 may be an upstream regulator of the ERK-P90RSK signaling pathway.

This research showed that SETD5 was clearly expressed in both the cytoplasm and nuclei of NSCLC specimens, while SETD5 expression in normal lung tissues was low. The expression of SETD5 was related to clinicopathological factors and poor OS. Taken together, these results indicated that SETD5 may be an oncogenic factor; this finding is supported by the oncogenic role of other SET domain protein family members [8, 10, 11], except SETD2, which was demonstrated to be a tumor suppressor in renal and breast carcinomas [12, 14–16, 29]. SETD4 is an oncoprotein that is localized to both the cytoplasm and nuclei [10], similar to SETD5 in the present study. Previous studies indicated that SETD5 expression was related to the prognosis of prostate and breast cancers [22–24], but this research is the first to indicate a correlation between SETD5 expression and NSCLC prognosis.

We found that SETD5 overexpression enhanced invasion and migration in NSCLC cells, while SETD5 suppression led to decreased invasion and migration. Poissonnier et al. [21] showed that miR126-5p abolished leukocyte transendothelial migration by suppressing SETD5. These studies indicated that SETD5 may be involved in the process of migration and invasion. This hypothesis is supported by the subsequent observation that SETD5 overexpression upregulated Snail and downregulated Zo-1. Indeed, Snail and Zo-1 are involved in EMT [30, 31]. EMT is the process by which epithelial cells lose their epithelial features and gain mesenchymal characteristics, leading to higher migratory abilities. High expression of Snail will lead to EMT and chemotherapy resistance [30]. Zo-1 is a tight junction protein that is involved in cell-cell interactions. Therefore, loss of Zo-1 will be associated with nonadherent cells that are free to migrate [31]. Snail upregulation could be responsible for the decrease in Zo-1 and the induction of EMT [32, 33]. Snail levels are modulated by numerous signaling pathway factors [33–36], and the exact molecular mechanisms responsible for the upregulation of Snail by SETD5 in the present study require additional study.

Nevertheless, the present study strongly suggests that SETD5 may upregulate Snail and downregulate Zo-1 by promoting the phosphorylation of ERK, which is supported by previous studies [34, 37–39]. SETD5 possesses a conserved SET domain and a PH domain. Previous studies showed that the SET domain was responsible for histone lysine methylation [5, 6, 13]. Lu et al. [40] demonstrated that the PH domain of MKK1 is responsible for modulating ERK expression [40]. The role of the SET and PH domains of SETD5 in the activation of p-ERK remains to be further explored in NSCLC.

## Conclusions

In conclusion, we found that the overexpression of SETD5 was associated with lymph node metastasis, advanced TNM stage, and poor prognosis in patients with NSCLC. SETD5 may promote the migration and invasion of NSCLC by enhancing the expression of Snail and inhibiting that of ZO-1. SETD5 may be an upstream regulator of the ERK-P90RSK signaling pathway. These results indicate that SETD5 could be a factor involved in the aggressiveness of NSCLC and a potential target for improving the prognosis of NSCLC patients. The limitations of this study include the limited number of patients and follow-up time. However, the study of SETD5 is not complete. We will continue to explore the molecular and biological functions of SETD5.

## Data Availability

All data generated or analyzed during this study are included in this article. The datasets used and/or analyzed during the current study are available from the corresponding author upon reasonable request.

## Abbreviations

IHC: Immunohistochemistry
NSCLC: Non-small cell lung cancer
OS: Overall survival
SETD5: SET domain containing 5
WHO: World Health Organization
